# Incidence of bone metastases in patients with solid tumors: analysis of oncology electronic medical records in the United States

**DOI:** 10.1186/s12885-017-3922-0

**Published:** 2018-01-06

**Authors:** Rohini K. Hernandez, Sally W. Wade, Adam Reich, Melissa Pirolli, Alexander Liede, Gary H. Lyman

**Affiliations:** 1 Amgen, Inc., One Amgen Center Drive, Thousand Oaks, CA 91320 USA; 2 Wade Outcomes Research and Consulting, 358 South 700 East, Suite B432, Salt Lake City, UT 84102 USA; 3 IMS Health, 1 IMS Drive, Plymouth Meeting, PA 19462 USA; 4 Fred Hutchinson Cancer Research Center, University of Washington School of Medicine, 1100 Fairview Ave N, Seattle, Washington, 98109 USA; 5 Amgen, Inc., 1120 Veterans Blvd, South San Francisco, CA 94114 USA

**Keywords:** Solid tumor, Bone metastasis, Incidence, Epidemiology

## Abstract

**Background:**

Bone metastases commonly occur in conjunction with solid tumors, and are associated with serious bone complications. Population-based estimates of bone metastasis incidence are limited, often based on autopsy data, and may not reflect current treatment patterns.

**Methods:**

Electronic medical records (OSCER, Oncology Services Comprehensive Electronic Records, 569,000 patients, 52 US cancer centers) were used to identify patients ≥18 years with a solid tumor diagnosis recorded between 1/1/2004 and 12/31/2013, excluding patients with hematologic tumors or multiple primaries. Each patient’s index date was set to the date of his or her first solid tumor diagnosis in the selection period. Kaplan-Meier analyses were used to quantify the cumulative incidence of bone metastasis with follow-up for each patient from the index date to the earliest of the following events: last clinic visit in the OSCER database, occurrence of a new primary tumor or bone metastasis, end of study (12/31/2014). Incidence estimates and associated 95% confidence intervals (CI) are provided for up to 10 years of follow-up for all tumor types combined and stratified by tumor type and stage at diagnosis.

**Results:**

Among 382,733 study patients (mean age 64 years; mean follow-up 940 days), breast (36%), lung (16), and colorectal (12%) tumors were most common. Mean time to bone metastasis was 400 days (1.1 years). Cumulative incidence of bone metastasis was 2.9% (2.9–3.0) at 30 days, 4.8% (4.7–4.8) at one year, 5.6% (5.5–5.6) at two years, 6.9% (6.8–7.0) at five years, and 8.4% (8.3–8.5) at ten years. Incidence varied substantially by tumor type with prostate cancer patients at highest risk (18% – 29%) followed by lung, renal or breast cancer. Cumulative incidence of bone metastasis increased by stage at diagnosis, with markedly higher incidence among patients diagnosed at Stage IV of whom11% had bone metastases diagnosed within 30 days.

**Conclusions:**

These estimates of bone metastasis incidence represent the experience of a population with longer follow-up than previously published, and represent experience in the recent treatment landscape. Underestimation is possible given reliance on coded diagnoses but the clinical detail available in electronic medical records contributes to the accuracy of these estimates.

## Background

Solid tumors frequently metastasize to bone [1, 2], and these bone metastases are associated with shortened survival [3–7] and increased risk of serious bone complications during the patients’ remaining lifespan [3, 8, 9]. Greater bone remodeling coincident with increased osteoblast and osteoclast activity at the site of the bone metastasis are hypothesized to create an environment that symbiotically supports tumor growth and bone destruction, and contributes to the risk of skeletal-related events (SREs) including pathological fractures and spinal cord compressions requiring palliative radiotherapy or surgery to bone [10, 11]. Patients with an SRE are significantly more likely to experience a subsequent SRE, often have a poorer prognosis and shorter overall survival than patients without an SRE, experience impaired quality of life, including ongoing pain, and consume significantly more health resources compared with patients without SREs [12–14].

Despite the important clinical and economic consequences of bone metastases, the incidence of bone metastases is not well understood as population-based estimates are limited in number and scope, often providing insights for a single tumor type or age group [4–6], or providing estimates on the basis of autopsy data that likely exceed the incidence of bone metastases that are formally diagnosed in routine patient care [15]. In addition, published estimates based on older data may not reflect survival trends under recent treatment advances [16], and long follow-up periods are also rarely reported in the literature.

The current study was conducted to estimate the incidence of bone metastases reflecting the more recent treatment landscape for patients with solid tumors. Specifically, we estimated the cumulative incidence proportion of clinically-identified bone metastases for all solid tumors combined and by tumor type using electronic medical records (EMR) data from oncology clinics in the United States (US). Results are presented for various time intervals during up to ten years of follow-up.

## Methods

Electronic medical records housed in the Oncology Services Comprehensive Electronic Records (OSCER) database were used to identify patients for this study. OSCER contains data from over 569,000 patients treated at 52 geographically-dispersed community and hospital-affiliated oncology practices in the US since 2004. This source population includes patients with health benefits through, Medicare, Medicaid, or commercial coverage, as well as patients who pay directly for their medical care. The Institutional Review Board of each oncology practice approved collaboration to contribute data to a large longitudinal electronic health records database; informed patient consent was waived per the US framework for retrospective noninterventional studies. Individual patient-level data were protected against breach of confidentiality consistent with the final Health Insurance Portability and Accountability Act (HIPAA) Security Rule from the US Department of Health and Human Services.

Patients included in the study population were at least 18 years old and a solid tumor diagnosis recorded between January 1, 2004 and December 31, 2013. Each patient’s index date was set to the date of his or her first solid tumor diagnosis during the patient selection period. This date represents the date of the definitive solid tumor diagnosis recorded in the electronic medical record at the patient’s oncologist’s office. Since most solid tumor patients will initiate their anti-cancer treatments with an oncologists, these are likely to be newly-treated patients.

As we sought to accurately assign patients to a primary solid tumor type based on the available data, we noted that a small percentage (2.3%) of patients had more than one primary tumor type recorded within 30 days of the index date. Therefore, the following rules were applied. Patients with multiple synchronous primaries (i.e., 3 or more different tumor types, including the index tumor) within 30 days of the index date were excluded. The following rules were used to determine the primary tumor type for patients with a second tumor type recorded within 30 days of the index tumor. Lung, liver, brain and bone tumors were considered to be metastases of the index tumor. Whenever present, melanoma was considered the primary solid tumor diagnosis. When both a non-specific and a specific tumor diagnosis code were present, the specific diagnosis defined the primary tumor type (e.g., gynecological cancer [non-specific] versus ovarian cancer [specific]). If two specific but different tumor types were recorded (including the index tumor diagnosis), the patient was excluded as the primary solid tumor type could not be clearly distinguished (e.g., breast cancer and colorectal cancer). Patients with only a non-specific tumor type in conjunction with a bone cancer diagnosis were also excluded. Since the primary study outcome was incident bone metastases (ICD-9 diagnosis code 198.5), patients with evidence of bone metastases more than 30 days prior to their index date were also excluded. Patients with bone metastases diagnosed within 30 days before their index date were considered to have bone metastasis at their initial solid tumor diagnosis, and the bone metastasis date was recoded to the index date.

Kaplan-Meier analyses were used to quantify the cumulative incidence of bone metastasis with follow-up for each patient from the index date to the earliest of the following events: last clinic visit in the OSCER database, occurrence of bone metastasis (including those diagnosed at index) or a new primary tumor, end of study (December 31, 2014). Incidence estimates and associated 95% confidence intervals (CI) are provided for up to 10 years of follow-up, with results for all tumor types combined and stratified by tumor type and stage at diagnosis.

## Results

**Fig. 1:**
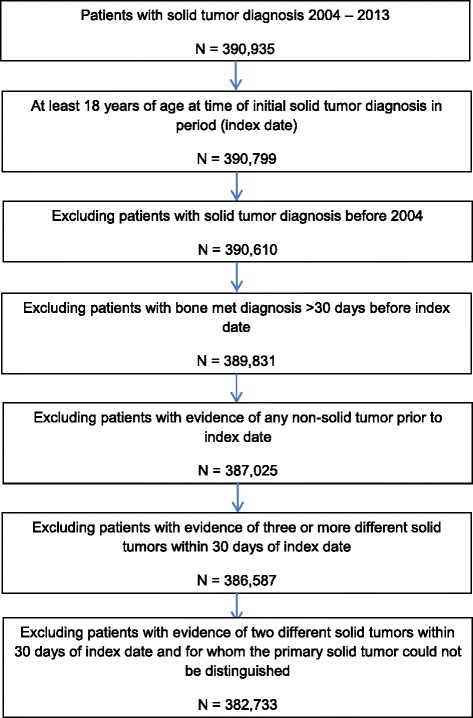
Selection of Study Patients

The majority (98%) of the 390,935 patients identified in OSCER with a new solid tumor diagnosis between January 1, 2004 and December 31, 2013 met all other selection criteria for inclusion in the study (Fig. 1). The most common reasons for exclusion were presence of non-solid tumor diagnoses (0.7%) and inability to determine the primary tumor type among patients with an additional tumor type recorded within 30 days prior to the index tumor as per the rules described in the methods section (1%).

Among the 382,733 study patients (mean age 64 years; mean follow-up 940 days), breast (36%), lung (16), and colorectal (12%) tumors were the most common index tumor types (Table 1). The number of patients identified in each year of the study increased from 16,525 in 2004 to 52,534 in 2013, with slightly more than half of study patients identified between 2010 and the end of 2013; this trend reflects the growing number of patients in the OSCER database in general.

**Table 1:** Demographic and clinical characteristics of study patients

Demographics	Total *N* (%)	Breast Cancer *N* (%)	Prostate Cancer *N* (%)	Lung Cancer *N* (%)	Colorectal Cancer *N* (%)	GI Cancers *N* (%)	Gynecological Cancers *N* (%)	Malignant Melanoma *N* (%)	Renal Cancers *N* (%)	All Other Solid Tumors *N* (%)
Total Number of Patients with Solid Tumor	382,733	137,720	22,801	59,344	46,832	32,874	21,075	12,152	17,717	32,218
Age Distribution										
18–35 Yrs Old	7115 (1.9)	1683 (1.2)	2 (<0.1)	102 (0.2)	560 (1.2)	296 (0.9)	731 (3.5)	606 (5.0)	1378 (7.8)	1757 (5.5)
35–49 Yrs Old	45,176 (11.8)	22,954 (16.7)	286 (1.3)	2524 (4.3)	4723 (10.1)	2528 (7.7)	3213 (15.2)	1851 (15.2)	2120 (12.0)	4977 (15.4)
50–59 Yrs Old	78,092 (20.4)	32,909 (23.9)	2349 (10.3)	9410 (15.9)	9425 (20.1)	6509 (19.8)	4781 (22.7)	2250 (18.5)	2746 (15.5)	7713 (23.9)
60+ Yrs Old	252,350 (65.9)	80,174 (58.2)	20,164 (88.4)	47,308 (79.7)	32,124 (68.6)	23,541 (71.6)	12,350 (58.6)	7445 (61.3)	11,473 (65.8)	17,771 (55.2)
Median Age	65.0	62.0	72.0	69.0	67.0	67.0	62.0	65.0	66.0	61.0
Gender										
Male	137,407 (35.9)	956 (0.7)	22,801 (100)	30,884 (52)	24,279 (51.8)	19,174 (58.3)	0	7040 (57.9)	12,866 (72.6)	19,407 (60.2)
Female	245,267 (65.1)	136,741 (99.3)	0	28,448 (47.9)	22,543 (48.1)	13,696 (41.7)	21,075 (100)	5111 (42.1)	4849 (27.4)	12,804 (39.7)
Stage										
Stage I	54,305 (14.2)	37,042 (26.9)	533 (2.3)	4955 (8.3)	2834 (6.1)	1424 (4.3)	3922 (18.6)	1581 (13.0)	1654 (9.3)	360 (1.1)
Stage II	47,080 (12.3)	27,886 (20.2)	2737 (12.0)	2603 (4.4)	7105 (15.2)	2998 (9.1)	1008 (4.8)	822 (6.8)	1297 (7.3)	624 (1.9)
Stage III	35,289 (9.2)	8720 (6.3)	591 (2.6)	7540 (12.7)	9303 (19.9)	2584 (7.9)	3012 (14.3)	877 (7.2)	1210 (6.8)	1452 (4.5)
Stage IV	45,527 (11.9)	5985 (4.3)	3908 (17.1)	13,487 (22.7)	7125 (15.2)	5840 (17.8)	2076 (9.9)	752 (6.2)	2668 (15.1)	3686 (11.4)
Unknown/Not Recorded	200,532 (52.4)	58,087 (42.2)	15,032 (65.9)	30,759 (51.8)	20,465 (43.7)	20,028 (60.9)	11,057 (52.5)	8120 (66.8)	10,888 (61.5)	26,096 (81.0)
Length of Follow Up Time (initial cancer diagnosis to last visit in database)										
Mean Days	940	1339	827	523	982	492	850	784	749	703

Of the full study population, 26,250 (6.9%) patients were diagnosed with bone metastases at index and during follow-up (median follow-up of 548 days [1.5 years] after the index solid tumor diagnosis).The mean time to bone metastasis from solid tumor diagnosis was 400 days (1.1 years), while the median time between diagnosis and bone metastasis was 69 days The corresponding intervals were 535 and 226 days after excluding patients with bone metastasis at index, and 700 and 407 days after excluding patients with bone metastasis within 30 days of their primary tumor diagnosis.

**Fig. 2:**
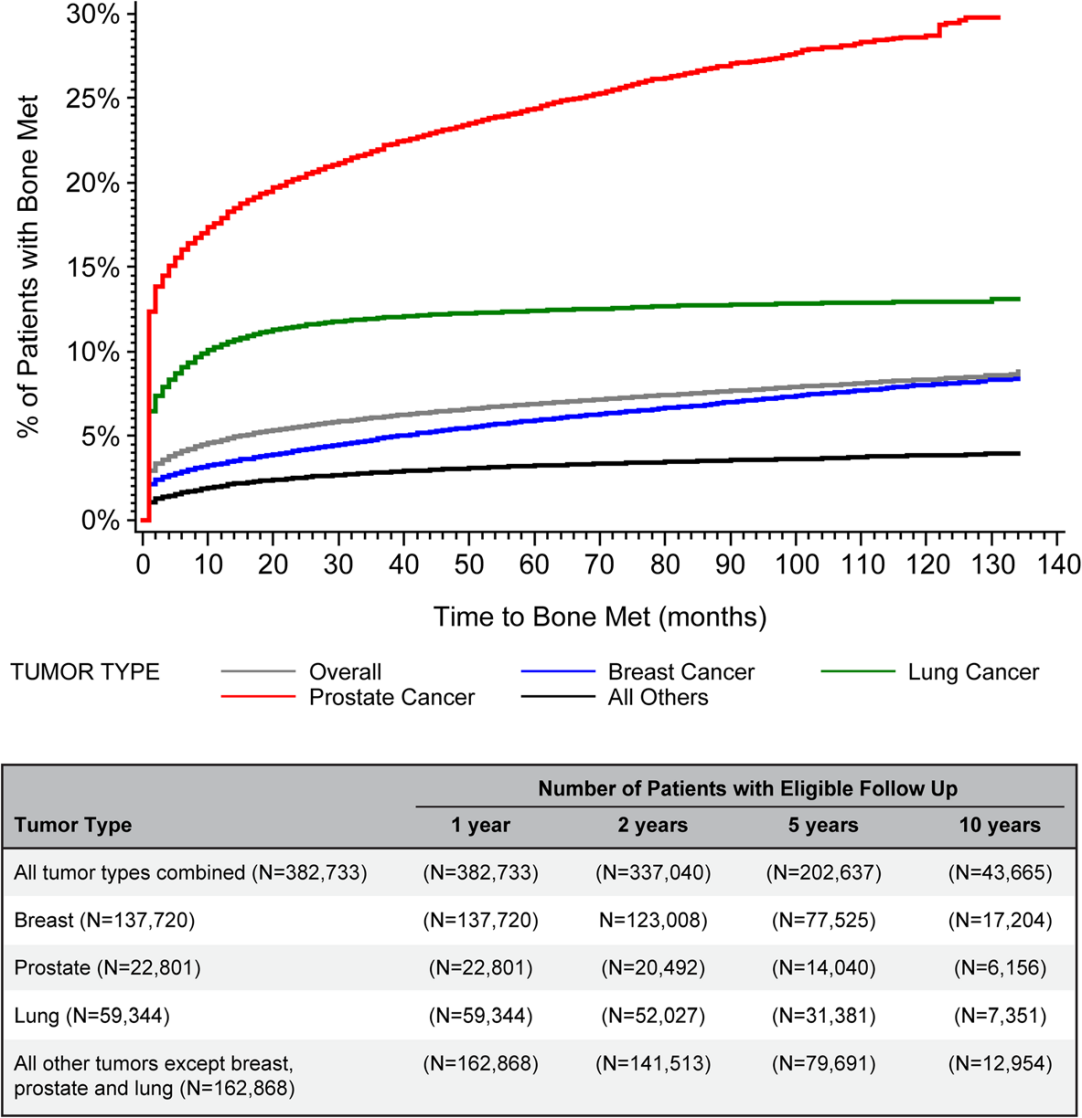
Cumulative bone metastasis incidence in 10-year follow-up of patients with solid tumors

For all tumor types combined, the cumulative incidence of bone metastasis (95% CI) was 2.9% (2.9–3.0) at 30 days post-index date, 4.8% (4.7–4.8) at one year, 5.6% (5.5–5.6) at two years, 6.9% (6.8–7.0) at five years, and 8.4% (8.3–8.5) at ten years (Fig. 2, Table 2). Bone metastasis incidence was highly variable depending on the primary tumor type, with prostate cancer patients at highest risk of developing bone metastases, followed by patients with lung, renal or breast cancer (Fig. 2, Table 2). The largest increase in incidence over the ten year follow-up was observed among patients with prostate cancer.

**Table 2:** 1-, 2-, 5-, and 10-year incidence of bone metastases by tumor type

Tumor type	Incidence of bone metastases (%)			
	1-year (95% CI)	2-year (95% CI)	5-year (95% CI)	10-year (95% CI)
All tumor types combined (*N* = 382,733)	4.8 (4.7–4.8)	5.6 (5.5–5.6)	6.9 (6.8–7.0)	8.4 (8.3–8.5)
Breast (*N* = 137,720)	3.4 (3.3–3.5)	4.2 (4.1–4.3)	6.0 (5.8–6.1)	8.1 (7.9–8.3)
Prostate (*N* = 22,801)	18.0 (17.5–18.5)	20.4 (19.9–20.9)	24.5 (23.9–25.1)	29.2 (28.3–30.1)
Lung (*N* = 59,344)	10.4 (10.2–10.7)	11.5 (11.3–11.8)	12.4 (12.1–12.7)	12.9 (12.6–13.2)
Colorectal (*N* = 46,832)	1.0 (0.9–1.1)	1.4 (1.3–1.5)	2.1 (2.0–2.3)	2.7 (2.5–2.9)
Gastrointestinal (*N* = 32,874)	2.3 (2.1–2.5)	2.7 (2.6–2.9)	3.2 (3.0–3.4)	3.6 (3.3–3.8)
Gynecological (*N* = 21,075)	1.1 (0.9–1.2)	1.3 (1.2–1.5)	1.9 (1.7–2.1)	2.4 (2.1–2.7)
Malignant melanoma (*N* = 12,152)	1.6 (1.4–1.8)	2.0 (1.7–2.2)	2.5 (2.2–2.8)	3.0 (2.6–3.4)
Renal (*N* = 17,717)	5.8 (5.5–6.2)	6.9 (6.6–7.3)	8.4 (8.0–8.9)	9.9 (9.3–10.5)
All other tumors (*N* = 32,218)	2.0 (1.8–2.1)	2.5 (2.3–2.7)	3.2 (3.0–3.4)	3.9 (3.5–4.2)

**Fig. 3:**
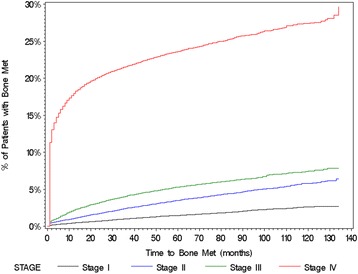
Cumulative bone metastasis incidence by stage at diagnosis for all solid tumors combined

The cumulative incidence of bone metastasis increased by stage at diagnosis, for the population overall (Fig. 3) and each tumor type (Table 3), with this pattern appearing in each follow-up interval assessed. In every case, the incidence of bone metastasis among patients diagnosed at Stage IV was markedly higher than incidence among patients diagnosed at less advanced disease stages. Although bone metastases were diagnosed on or within 30 days of the solid tumor diagnosis in 11% (5206/45,527) of the patients who were diagnosed at Stage IV, the cumulative incidence of bone metastasis continued to increase in these late-stage patients over time, regardless of tumor type.

**Table 3:** 1-, 2-, 5-, and 10-year incidence of bone metastases by tumor type and stage at diagnosis

Tumor type	Incidence of bone metastases (%)			
	1-year (95% CI)	2-year (95% CI)	5-year (95% CI)	10-year (95% CI)
All tumor types combined (N = 382,733)				
Stage I (*N* = 54,305)	0.5 (0.4–0.5)	0.7 (0.7–0.8)	1.5 (1.4–1.6)	2.7 (2.4–2.9)
Stage II (*N* = 47,080)	1.1 (1.0–1.2)	1.8 (1.7–1.9)	3.6 (3.4–3.8)	5.9 (5.6–6.3)
Stage III (*N* = 35,289)	2.2 (2.0–2.3)	3.3 (3.1–3.5)	5.4 (5.1–5.6)	7.5 (7.1–8.0)
Stage IV (*N* = 45,527)	18.0 (17.7–18.4)	20.3 (19.9–20.7)	23.7 (23.3–24.1)	27.6 (27.0–28.2)
Breast (*N* = 137,720)				
Stage I (*N* = 37,042)	0.3 (0.3–0.4)	0.5 (0.5–0.6)	1.2 (1.1–1.4)	2.4 (2.1–2.7)
Stage II (*N* = 27,886)	1.0 (0.9–1.2)	1.8 (1.6–1.9)	3.9 (3.7–4.2)	6.5 (6.0–6.9)
Stage III (*N* = 8720)	2.9 (2.6–3.3)	4.9 (4.4–5.3)	10.1 (9.4–10.8)	15.3 (14.1–16.5)
Stage IV (*N* = 5985)	36.4 (35.2–37.7)	41.4 (40.2–42.7)	50.6 (49.3–52.0)	61.4 (59.5–63.2)
Prostate (*N* = 22,801)				
Stage I (*N* = 533)	3.0 (1.8–4.9)	4.4 (2.9–6.5)	7.7 (5.4–10.8)	12.1 (8.3–17.4)
Stage II (*N* = 2737)	3.3 (2.6–4.0)	4.1 (3.4–4.9)	7.3 (6.3–8.4)	15.8 (12.0–20.6)
Stage III (*N* = 591)	9.0 (6.9–11.6)	11.5 (9.2–14.4)	16.4 (13.4–19.9)	23.4 (18.7–29.0)
Stage IV (*N* = 3908)	45.3 (43.8–46.9)	51.4 (49.9–53.0)	61.1 (59.5–62.8)	70.7 (68.4–72.9)
Lung (N = 59,344)				
Stage I (*N* = 4955)	1.4 (1.1–1.8)	2.3 (1.9–2.7)	3.6 (3.0–4.2)	5.1 (4.1–6.4)
Stage II (*N* = 2603)	2.7 (2.2–3.4)	4.2 (3.4–5.0)	5.6 (4.7–6.7)	8.4 (6.9–10.4)
Stage III (*N* = 7540)	4.4 (4.0–4.9)	5.8 (5.3–6.4)	6.7 (6.2–7.3)	7.4 (6.7–8.2)
Stage IV (N = 13,487)	22.9 (22.2–23.6)	24.5 (23.8–25.3)	25.8 (25.1–26.6)	26.2 (25.4–27.0)
Colorectal (*N* = 46,832)				
Stage I (*N* = 2834)	0.2 (0.1–0.5)	0.4 (0.2–0.7)	1.0 (0.7–1.5)	1.6 (1.0–2.6)
Stage II (*N* = 7105)	0.2 (0.1–0.3)	0.5 (0.3–0.7)	1.0 (0.7–1.3)	1.6 (1.1–2.2)
Stage III (*N* = 9303)	0.4 (0.3–0.5)	0.7 (0.6–0.9)	1.5 (1.2–1.8)	1.9 (1.6–2.3)
Stage IV (*N* = 7125)	3.0 (2.7–3.5)	4.1 (3.7–4.6)	5.8 (5.2–6.4)	6.6 (5.9–7.4)
Gastrointestinal (*N* = 32,874)				
Stage I (*N* = 1424)	0.4 (0.2–0.9)	0.7 (0.3–1.3)	1.5 (0.9–2.5)	2.2 (1.2–3.9)
Stage II (*N* = 2998)	0.7 (0.5–1.1)	1.3 (0.9–1.7)	2.2 (1.7–2.9)	2.9 (2.0–4.1)
Stage III (*N* = 2584)	1.2 (0.9–1.7)	1.9 (1.4–2.5)	2.7 (2.1–3.4)	2.9 (2.2–3.7)
Stage IV (*N* = 5840)	5.3 (4.7–5.9)	6.1 (5.5–6.8)	6.8 (6.2–7.5)	7.4 (6.5–8.4)
Gynecological (*N* = 21,075)				
Stage I (*N* = 3922)	0.2 (0.1–0.4)	0.5 (0.3–0.8)	0.9 (0.6–1.3)	1.9 (1.1–3.2)
Stage II (*N* = 1008)	0.6 (0.3–1.3)	1.0 (0.5–1.9)	1.8 (1.1–3.0)	2.0 (1.3–3.3)
Stage III (*N* = 3012)	0.7 (0.4–1.0)	1.2 (0.9–1.7)	2.1 (1.6–2.8)	3.1 (1.9–5.1)
Stage IV (*N* = 2076)	4.0 (3.3–5.0)	4.7 (3.8–5.7)	6.2 (5.2–7.5)	7.5 (5.9–9.6)
Malignant melanoma (N = 12,152)				
Stage I (*N* = 1581)	0.2 (0.1–0.6)	0.3 (0.1–0.7)	0.6 (0.3–1.3)	0.9 (0.4–1.7)
Stage II (*N* = 822)	0.7 (0.3–1.6)	1.1 (0.6–2.1)	1.9 (1.1–3.3)	1.9 (1.1–3.3)
Stage III (*N* = 877)	0.6 (0.2–1.4)	1.2 (0.6–2.1)	1.2 (0.6–2.1)	1.2 (0.6–2.1)
Stage IV (*N* = 752)	6.1 (4.6–8.1)	7.4 (5.7–9.5)	9.0 (7.1–11.5)	10.4 (8.0–13.5)
Renal (*N* = 17,717)				
Stage I (*N* = 1654)	0.8 (0.5–1.4)	1.1 (0.7–1.8)	2.4 (1.7–3.4)	5.1 (3.4–7.6)
Stage II (*N* = 1297)	1.5 (1.0–2.4)	2.4 (1.7–3.4)	3.9 (2.9–5.3)	5.0 (3.5–7.1)
Stage III (*N* = 1210)	2.1 (1.5–3.1)	3.3 (2.4–4.5)	5.0 (3.8–6.5)	6.8 (5.1–9.1)
Stage IV (*N* = 2668)	15.5 (14.2–16.9)	18.3 (16.9–19.8)	22.3 (20.7–24.1)	26.2 (23.6–29.0)
Other Tumors (*N* = 162,868)				
Stage I (*N* = 360)	0.8 (0.3–2.6)	1.7 (0.8–3.7)	2.1 (1.0–4.4)	5.2 (1.6–16.3)
Stage II (*N* = 624)	0.8 (0.3–1.9)	1.3 (0.7–2.6)	3.2 (2.0–5.3)	4.8 (2.9–7.8)
Stage III (*N* = 1452)	0.8 (0.4–1.4)	1.5 (1.0–2.3)	1.9 (1.3–2.8)	3.5 (2.1–5.7)
Stage IV (*N* = 3686)	2.7 (2.2–3.3)	3.8 (3.2–4.5)	4.6 (4.0–5.4)	5.3 (4.3–6.4)

## Discussion

This study estimated the cumulative incidence of bone metastasis among patients with solid tumors using real world electronic medical record data from oncology practices in the US. To our knowledge, this is the first large-scale US study to estimate the incidence of bone metastases for all solid tumors combined and by tumor type, with patients followed for up to 10 years after their initial solid tumor diagnosis. Cumulative incidence increased from 2.9% within 30 days of the first solid tumor diagnosis in the study period to 8.4% during a ten year follow-up period. Bone metastasis incidence increased most quickly in the first two years for the solid tumor population as a whole, with the most common tumor types also showing the greatest increases in incidence in the first year or two post-diagnosis. The availability of long-term follow-up data for the study population allowed us to determine that the cumulative incidence of bone metastasis also continued increasing for at least ten years after the initial solid tumor diagnosis, regardless of tumor type.

In our study population, patients with prostate tumors exhibited markedly higher incidence of bone metastases in every time interval assessed, and substantially larger increases in incidence from the first through tenth year of follow-up. It is important to note that the sample of prostate cancer patients with data in OSCER includes only patients who were treated at a participating oncology clinic. This approach may bias our sample toward men with later stage disease, compared with the general prostate cancer population, by excluding men who received their prostate cancer care in urology clinics. These excluded patients would presumably be more likely to have early stage disease and a generally lower propensity for disease progression including the development of bone metastases over time. If early stage patients are under-represented in our sample, as we expect, our results likely exceed the true bone metastasis incidence in a more typical prostate cancer population. Although early stage prostate cancer patients may be less well-represented in our population, the observed trend in incidence over time suggests that ongoing monitoring of bone health may continue to be important for patients with prostate cancer, even years after the initial prostate cancer diagnosis. Surprisingly, the literature suggests that such monitoring to identify an initial bone metastasis is not generally routine with one study reporting that even prostate cancer patients at high risk of developing bone metastases, such as those with prostate-specific antigen doubling time less than 3 months, did not routinely receive a second bone scan within one year after a first negative bone scan [17]. There is not yet a universal guideline regarding imaging of men with M0 castration-resistant prostate cancer, but appropriate screening frequency will need to balance the potential benefits that could be obtained through early detection and treatment with cost considerations [17].

Not surprisingly, we found that the incidence of bone metastasis was higher among patients with more advanced disease (i.e., higher stage) at diagnosis in the solid tumor population overall and for the individual tumor types that we examined. This pattern continued over time; we noted this relationship between stage at diagnosis and bone metastasis incidence in every follow-up interval for the study population overall and for each tumor type. Greater incidence of bone metastases among patients with higher cancer stages at diagnosis has also been reported previously in population-based studies of breast cancer patients in Denmark and the United Kingdom (UK) [16, 18].

The literature on bone metastasis incidence in the US provides estimates for three important tumor types, but only for individuals with Medicare coverage whose administrative claims data could be linked to data in the population-based Surveillance Epidemiology and End Results (SEER) cancer registry [4–6]. Using these data, Sathiakumar et al. have reported separately on the experience of patients diagnosed with lung, breast or prostate cancer between 1999 and 2005 and followed through the end of study in 2006. These studies were limited to patients age 65 and older and to those individuals who had full fee-for-service Medicare coverage for at least 6 months prior to their cancer diagnosis. In addition, these older data do not reflect changes in survival and disease progression stemming from recent improvements to the treatment landscape. Although differences in the underlying populations and prevailing treatment regimens preclude direct comparisons, these earlier studies provide useful context for our tumor-specific findings. The reported cumulative incidence proportions at diagnosis and follow-up, respectively, were 7.6% and 12.1% for lung cancer (median follow-up 0.6 years), 1.5% and 5.8% for breast cancer (median follow-up 3.3 years), and 1.7% and 5.9% for prostate cancer (median follow-up 3.3 years).

**Fig. 4:**
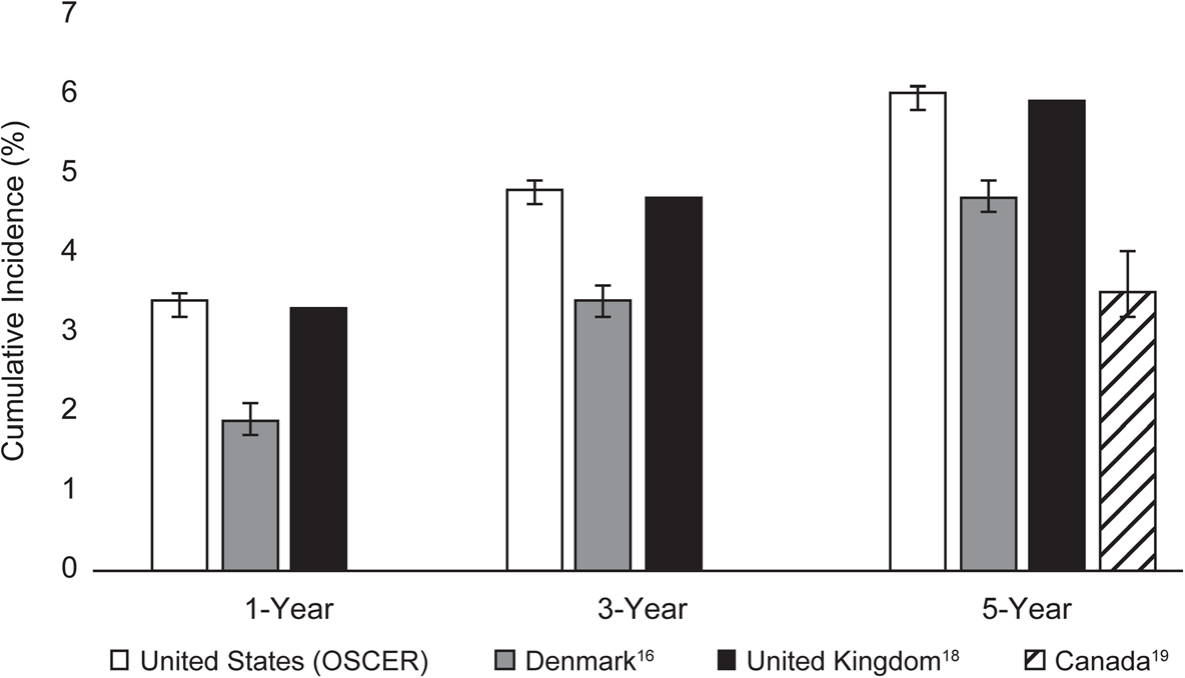
Country-specific cumulative bone metastasis incidence estimates for women with breast cancer

Population-based estimates of the incidence of bone metastasis among patients with breast cancer have been reported for populations in Canada [19], the UK [18], and Denmark where survival after bone metastasis and related complications (SREs) has also been assessed [3, 7, 16, 20, 21] (Fig. 4). The Canadian study, which reports the experience of women diagnosed with non-metastatic breast cancer between 1989 and 2001, reports on trends in the incidence of bone metastases over time. The 5-year incidence of bone metastasis underwent a continuous decrease (7.46% [95% CI: 6.66, 8.31], 5.25% [95% CI: 4.80, 5.71] and 3.54% [95% CI: 3.16, 3.96] in cohorts diagnosed between 1989–1991, 1992–1997, and 1998–2001. These cohorts were constructed to reflect important evolutions in the breast cancer treatment landscape. Specifically, in the first cohort, first generation CMF (cyclophosphamide, methotrexate, and 5 fluorouracil) chemotherapy without hormone therapy was used for premenopausal women with node positive cancers or high-risk node negative tumors. Postmenopausal women received tamoxifen regardless of tumor hormonal status, with those at high-risk also receiving 6 cycles of an anthracycline-containing regimen. The second cohort would have experienced increased tamoxifen use for premenopausal women and greater anthracycline-based chemotherapy for both pre- and postmenopausal women. The third cohort would have seen greater use of adjuvant anthracyclines with the introduction of taxane and aromatase inhibitors in patients with either estrogen receptor positive (ER+) or estrogen receptor negative (ER-) tumors.

In the UK study, the authors examined the experience of 13,207 women diagnosed with breast cancer between 2000 and 2006, using data from General Practice Research Database (GPRD) linked to the National Cancer Registry (NCR) and Hospital Episode Statistics (HES) [18]. In this population, most women had Stage 1 or 2 disease at diagnosis, but 2.6% of patients had metastatic breast cancer at diagnosis. After a median follow-up of 5.4 years, 6% of patients had developed bone metastases. The cumulative incidence of bone metastasis ranged from 3.3% at one year to 5.9% at five years. Another smaller scale UK study examined the occurrence of distance metastases in women treated for primary invasive breast cancers at two National Health Service Trust Foundation hospitals between 1975 and 2006 [22]. The five year cumulative incidence of bone metastases was estimated at 6.9% (95% CI, 6.3–7.5) among women with unilateral breast cancer, 11% (95% CI, 5.1–16) among women with metachronous contralateral breast cancer occurring within five years of the initial diagnosis, and 2.3% (95% CI, 0.06–4.6) among women with metachronous contralateral breast cancer occurring more than five years after the initial diagnosis.

The population-based studies in Denmark used data from the Danish National Patient Registry (DNPR), which includes data from all hospitals in the country, to examine the incidence of bone metastases separately for patients with diagnosed with breast, lung and prostate cancer from 1999 through 2007. For a cohort of female breast cancer patients, Jensen et al. reported that the cumulative incidence of bone metastases increased from 1.9% (1.7–2.0) at one year to 3.4% (3.2–3.6) at three years to 4.7% (4.4–4.9) at five years [16]. In the prostate cancer cohort, the cumulative incidence of bone metastasis at one and five years after diagnosis was 7.7% (7.4–8.1) and 16.6% (95% CI 16.0–17.1), respectively [3]. In the lung cancer cohort, the cumulative incidence of bone metastases was 5.9% (5.6–6.2) at one year and 6.7% (6.4–7.0) at three years [20].

Development of bone metastases is an important prognostic indicator, with population-based studies demonstrating a significantly shorter survival after bone metastases occur. [4–7, 21] SREs may play an important role in the increased mortality risk subsequent to the development of bone metastases. Norgaard et al., for example, note that fewer than 1% of prostate cancer patients with bone metastases and SREs survived five years after their diagnosis [3], and suggest that SREs may signify more advanced or aggressive disease that shorten survival, and as other researchers have indicated [23], surgery for pathological fracture and loss of mobility and functional independence may also contribute to increased mortality [3].

Since 1996, three agents have been marketed in the US for the prevention of SREs in patients with bone metastasis secondary to solid tumors (intravenous bisphosphonates [IVBP]: zoledronic acid (4 mg) and pamidronate disodium, dosed every 3–4 weeks; denosumab 120 mg, a RANK ligand inhibitor dosed every 4 weeks). With effective treatment options available and evidence regarding the significant mortality and morbidity implications of bone metastasis and SREs accumulating in the medical literature, bone health is increasingly addressed in key clinical guidelines [24, 25]. Even with this increased attention, one recent study of solid tumor patients with bone metastases in the US found that only 43% of commercially-insured patients and 47% of patients with Medicare coverage received bone targeted agents in 2012 [26]. Furthermore, over half of these patients (53% commercial, 57% Medicare) initiated these agents only after experiencing a bone complication. This finding is especially concerning in light of results from a recent study of breast cancer patients suggesting that the timing of bone targeting agent initiation has potential to significantly shape the level of therapeutic benefit to the patient [9]. In that study, the risk and frequency of SREs was higher if bone modifying agents (BMA) were not initiated until ≥6 months after bone metastasis diagnosis. Additionally, the presence of extraskeletal metastases was associated with shorter time to first SRE.

Study limitations include access only to patients who received treatment or were under active surveillance at one of the OSCER-contributing clinics. Although this population includes patients with a variety of solid tumors, the tumor type distribution in our study differs from that in the U.S. population overall. Thus, the incidence estimate for the overall solid tumor category in our study may not be generalizable to the U.S. population. Specifically, patients with breast cancer may continue seeing their oncologists long after completing their active cancer treatment, and therefore, may be over-represented in the OSCER database. Prostate cancer patients overall, and early-stage patients in particular, may be under-represented in the study population, since many such patients are cared for exclusively at urology clinics. Estimates of bone metastasis incidence for all solid tumors combined are reported here for completeness and to provide context for the tumor-specific incidence estimates that we report. Our reliance on coded bone metastasis diagnoses may result in a conservative estimate of incidence. A recent study examining the validity of bone metastasis capture in the OSCER database found high specificity (98%) and lower sensitivity (67%) which provides reassurance that identified cases are true cases, yet suggests that identification of bone metastasis cases is not complete using the structured EMR data captured in OSCER [27]. Examination of the timing of bone metastasis coding suggested that the decision to treat (e.g., prescribing of a bone targeting agent or referral to orthopedic surgeon or radiation oncologist) may trigger the formal recording of a bone metastasis diagnosis. More generally, such misclassification is a limitation in all real-world databases used to estimate bone metastasis incidence [4–6], although the earlier validation study indicates that OSCER-based analyses are likely to better capture bone metastasis compared with analyses that use administrative claims data [28]. Ultimately, chart review remains the gold standard for case identification, but is feasable only for studies with small populations or limited follow-up, given the costs and records access required. Unlike such small-scale studies, our study provided access to EMR data for a large and diverse population of solid tumor patients in which we estimated the incidence of bone metastases during up to ten years of follow. In contrast to the potential underestimation associated with coding considerations, our incidence estimates include bone metastases that occurred around the index date (i.e., at index or within 30 days of index which can be interpreted as prevalent bone metastases) and this approach has the potential for overestimation. As expected, these early bone metastases were more likely to occur in patients with more advanced disease at diagnosis, and, although data on stage at diagnosis were limited (52% missing) for the study population, half of the patients with bone metastases at or within 30 days of index were classified as Stage IV at diagnosis. Stage data is likely missing at random, since tumors are typically staged at the initial diagnosis, and these data are not routinely recorded in the structured portions of the electronic medical records. Although the true incidence of bone metastases may differ from our estimates, these results provide useful insights into bone metastasis occurrence and trends in the current treatment landscape.

## Conclusions

In summary, our study estimated the incidence of bone metastases for solid tumor patients in the US, with 1-, 2-, 5- and 10-year estimates provided for solid tumors in aggregate, for individual tumor types, and by stage at diagnosis. Unique strengths of the study are the inclusion of all solid tumor types within a demographically and geographically broad population (no age or insurance type restrictions, a large population treated at over 52 oncology practices across the US), a follow-up period which is substantially longer than previously published incidence studies, and visibility into incidence shaped by current treatment approaches.

## Abbreviations

BMA: bone modifying agents
CI: confidence interval
CMF: cyclophosphamide, methotrexate, and 5 fluorouracil
DNPR: Danish National Patient Registry
EMR: electronic medical records
ER: estrogen receptor
FDA: US Food and Drug Administration
GPRD: General Practice Research Database
HES: Hospital Episode Statistics
ICD-9: International Classification of Diseases, 9th Revision
IVBP: intravenous bisphosphonates
NCR: National Cancer Registry
OSCER: Oncology Services Comprehensive Electronic Records
SEER: Surveillance Epidemiology and End Results
SRE: skeletal-related event
UK: United Kingdom
US: United States
